# Knockdown of CDK2AP1 in Primary Human Fibroblasts Induces p53 Dependent Senescence

**DOI:** 10.1371/journal.pone.0120782

**Published:** 2015-03-18

**Authors:** Khaled N. Alsayegh, Venkat S. Gadepalli, Shilpa Iyer, Raj R. Rao

**Affiliations:** 1 Department of Human and Molecular Genetics, Virginia Commonwealth University, Richmond, United States of America; 2 King Abdullah International Medical Research Center, King Saud bin Abdulaziz University for Health Sciences, Jeddah, Saudi Arabia; 3 Integrated Life Sciences Program, Virginia Commonwealth University, Richmond, Virginia, United States of America; 4 Center for the Study of Biological Complexity, Life Sciences Program, Virginia Commonwealth University, Richmond, Virginia, United States of America; 5 Department of Chemical and Life Sciences Engineering, Virginia Commonwealth University, Richmond, Virginia, United States of America; 6 Massey Cancer Center, Virginia Commonwealth University, Richmond, Virginia, United States of America; Stony Brook University, UNITED STATES

## Abstract

Cyclin Dependent Kinase-2 Associated Protein-1 (CDK2AP1) is known to be a tumor suppressor that plays a role in cell cycle regulation by sequestering monomeric CDK2, and targeting it for proteolysis. A reduction of CDK2AP1 expression is considered to be a negative prognostic indicator in patients with oral squamous cell carcinoma and also associated with increased invasion in human gastric cancer tissue. CDK2AP1 overexpression was shown to inhibit growth, reduce invasion and increase apoptosis in prostate cancer cell lines. In this study, we investigated the effect of CDK2AP1 downregulation in primary human dermal fibroblasts. Using a short-hairpin RNA to reduce its expression, we found that knockdown of CDK2AP1in primary human fibroblasts resulted in reduced proliferation and in the induction of senescence associated beta-galactosidase activity. CDK2AP1 knockdown also resulted in a significant reduction in the percentage of cells in the S phase and an accumulation of cells in the G1 phase of the cell cycle. Immunocytochemical analysis also revealed that the CDK2AP1 knockdown significantly increased the percentage of cells that exhibited γ-H2AX foci, which could indicate presence of DNA damage. CDK2AP1 knockdown also resulted in increased mRNA levels of *p53*, *p21*, *BAX* and *PUMA* and p53 protein levels. In primary human fibroblasts in which p53 and CDK2AP1 were simultaneously downregulated, there was: (a) no increase in senescence associated beta-galactosidase activity, (b) decrease in the number of cells in the G1-phase and increase in number of cells in the S-phase of the cell cycle, and (c) decrease in the mRNA levels of *p21*, *BAX* and *PUMA* when compared with CDK2AP1 knockdown only fibroblasts. Taken together, this suggests that the observed phenotype is p53 dependent. We also observed a prominent increase in the levels of ARF protein in the CDK2AP1 knockdown cells, which suggests a possible role of ARF in p53 stabilization following CDK2AP1 knockdown. Altogether, our results show that knockdown of CDK2AP1 in primary human fibroblasts reduced proliferation and induced premature senescence, with the observed phenotype being p53 dependent.

## Introduction

CDK2AP1 is a cell cycle regulator that controls the G1-S phase transition by negatively regulating CDK2 [1]. In vitro studies focused on overexpression of CDK2AP1 in prostate cancer cell lines results in a decrease in levels of CDK2 and its kinase activity, leading to an accumulation of cells in the G1 phase and a reduction in cells that are in the S phase of the cell cycle [2]. This outcome has been reasoned to be mediated by either the sequestration of monomeric CDK2 or by targeting it for proteolysis. Another mechanism by which CDK2AP1 regulates G1-S phase transition, is by directly binding the DNA polymerase/alpha-primase complex and inhibiting the initiation step of DNA replication [3]. This inhibition may also be a result of CDK2AP1-mediated reduction in CDK2 activity, which is known to stimulate DNA replication by phosphorylating the DNA polymerase-alpha-primase complex.

CDK2AP1 has also been found to mediate the growth inhibitory effects of TGF-β with studies in normal human keratinocytes treated with TGF-β, increased cellular levels of CDK2AP1 mRNA and protein [4]. Analysis of the results suggests that SMAD induced by TGF-β1 binds at the proximal promoter of the CDK2AP1 gene. A significant correlative expression of TGF-β receptor II (TGFβRII) and CDK2AP1 has been found in human oral squamous cell carcinoma (OSCC) tissues with an observed loss of expression of CDK2AP1 and p21 [5]. It has also been found that OSCC lines that were resistant to TGF-β, were unable to induce SMADs and CDK2AP1, indicating a critical role for CDK2AP1 in mediating the growth inhibitory effects of TGF-β [5]. The effects of overexpressing CDK2AP1 in prostate cancer cell lines, in which it is downregulated were also evaluated [2]. Overexpression of CDK2AP1 in prostate cancer cell lines lead to increased apoptosis, growth arrest and reduced invasion. In gastric cancer, it was found that patients who had higher levels of CDK2AP1 in their samples had a better prognosis than patients who had low levels of CDK2AP1 [6]. Although the previously mentioned studies demonstrated the anti-tumorigenic role of CDK2AP1, a recent study revealed that knockdown of CDK2AP1 in human glioma inhibited growth and tumorigenesis [7]. It was shown that RNAi-mediated knockdown of CDK2AP1 in U251 and U373 human glioma cells resulted in reduction in cell proliferation and arrested cells in G0/G1 phase of the cell cycle. Furthermore, when xenograft formation was used to examine in vivo tumorigenesis, CKD2AP1downregulation was found to inhibit tumor growth [7].

In this study, we aimed to investigate the effect of CDK2AP1 knockdown in normal primary human dermal fibroblasts and demonstrate that knockdown of CDK2AP1 in these cells resulted in reduced proliferation and p53-dependent senescence.

## Materials and Methods

### Generation of primary human fibroblasts expressing CDK2AP1-specific shRNA and p53-specific shRNA

Primary human dermal fibroblasts (HDF) (Coriell Cell Repositories, NJ) were routinely maintained in medium containing MEM, 15% FBS, 100 U/ml penicillin and 100 μg/ml streptomycin, with subculturing ratios of 1:4 using 0.05% Trypsin solution. All reagents were obtained from Invitrogen (Carlsbad, CA) unless otherwise noted.

We have identified two potent shRNAs targeted to CDK2AP1 mRNA. Multiple shRNAs were obtained from commercially available sources (Open Biosystems, PA; Sigma-Aldrich, MO) and screened for their effectiveness. Control scrambled sequences were used similarly. To identify the shRNA clone that produced the strongest knockdown of CDK2AP1, human fibroblasts were transduced with the different shRNA clones using lentiviral vectors, and successfully transduced cells were selected by puromycin treatment (3 μg/ml). Following 6 days of selection, antibiotic-resistant cells were harvested and RNA extracted. QPCR analyses of human CDK2AP1specific primers were conducted. In our experiments, one shRNA (labeled as shRNA1 henceforth) (Open Biosystems, PA) produced the strongest knockdown and was used in subsequent experiments. A second validated CDK2AP1 shRNA (labeled as shRNA2 henceforth) (Sigma-Aldrich, MO) that target the 3’-UTR, was also used in our studies. In experiments involving analysis of the role of p53, expression was downregulated using lentiviral delivery of p53-specific shRNA (Addgene, MA, USA), followed by validation of knockdown by qPCR analyses.

### RNA isolation, Real Time Reverse Transcription Polymerase Chain Reaction (RT-PCR), and gene expression analysis

RNA isolation and gene expression analysis was performed as described previously [8]. Briefly, RNA was isolated using RNeasy kit (Qiagen, CA, USA), according to the manufacturer’s protocols and quantified using BioMate3 UV-VIS Spectrophotometer (Thermo Scientific, MA, USA). Complimentary DNA (cDNA) was synthesized from 1 μg of RNA using cDNA reverse transcription kit (Applied Biosystems, CA). Gene expression within different samples was analyzed using quantitative real time RT-PCR (QPCR). QPCR was performed in an ABI HT7900 system (Applied Biosystems, CA) and the data were acquired using sequence detection system software (SDS v2.2.1, Applied Biosystems, CA). Gene expression data (three replicates) were acquired and SDS software was used to estimate differential gene expression using ΔCT quantification methods. Endogenous *GAPDH* was used for normalization. Commercially available primers for *CDK2AP1* were obtained from Origene, MD, while other primers summarized in S1 Table were obtained from Integrated DNA Technologies, IA.

### Antibodies and Immunocytochemical analysis

Immunocytochemistry was performed as described previously [8,9]. In summary, human fibroblasts were seeded onto four chambered glass slides. Paraformaldehyde (PFA, 4%) in PBS was used for fixation, permeabilization for intracellular markers was achieved with 0.2% Triton X-100 in PBS and blocked with normal goat serum at room temperature. Fixed cells were incubated with primary antibodies: CDK2AP1 (Santa Cruz, CA), Anti-phospho-Histone H2AX (Millipore, CA). Goat anti-mouse IgG conjugated to Alexa 488 (for γ-H2AX staining) and Goat anti-rabbit IgG conjugated to Alexa 594 (for CDK2AP1 staining) (Invitrogen, CA,) were used as secondary antibodies. The samples were prepared for imaging by overnight exposure to secondary antibody at 4°C followed by PBS washes, nuclear stain using DAPI (4^’^,6-diamidino-2-phenylindole) and finally mounting the slides. Fluorescent images were acquired using a CoolSnap EZ camera (Photometrics, Tucson, AZ) mounted on a Nikon Eclipse TE 2000-S inverted microscope (Nikon, Melville, NY) with attached image analysis software. All image settings were controlled for uniform acquisition between samples. Specifically, a uniform exposure time was maintained for images acquired from experimental samples as well as negative controls for background subtraction. Percentages of γ-H2AX positive cells were calculated in multiple fields per sample (n = 28) by counting the number of cells containing at least 3 foci in their nucleus and dividing by the total number of cells in each field of interest.

### Bromodeoxyuridine (BrdU) proliferation assay

For proliferation assays, fibroblasts under different experimental conditions in four-chambered glass slides were propagated overnight, with subsequent incubation in medium containing 20 μg/ml Bromodeoxyuridine (BrdU) for a period of 2 hrs. Cells were then washed with ice cold PBS buffer and fixed with 100% methanol at -20°C for 15 min. After fixation, cells were washed in cold PBS and permeabilization achieved with 0.2% Triton X-100 in PBS. For DNA denaturation (antigen retrieval), cells were treated with 2N HCl solution for 45 min at room temperature. To neutralize the HCl solution, Borate buffer was added for 6 min and cells washed. Blocking was achieved by treatment with 3% goat serum in medium for 45 min at room temperature. Fixed cells were incubated with Anti-BrdU (Abcam, MA) as the primary antibody. Goat anti-rat IgG conjugated to Alexa 594 (Invitrogen, CA) was used as a secondary antibody. The samples were prepared for imaging by overnight exposure to secondary antibody at 4°C followed by PBS washes, nuclear stain using DAPI (4^’^,6-diamidino-2-phenylindole) and finally mounting the slides. To determine the percentage of BrdU positive cells, slides were viewed at 20X magnification and random fields chosen in which total number of cells was determined by counting DAPI, followed by counting the BrdU-positive cells. Triplicate counts of at least 500 cells each were acquired and the percentage of BrdU positive cells was determined by dividing the number of BrdU positive cells by total number of cells.

### Cell cycle profile analysis

Cell cycle analysis was performed as described previously [10]. Cells to be analyzed were trypsinized, washed, stained with propidium iodide for 45 min at 37°C, filtered through a 30 μm mesh to eliminate clumps and subjected to cell cycle analysis on an Accuri C6 Flow Cytometer (BD Biosciences, CA). Data were analyzed using the software provided by the manufacturer and samples analyzed in triplicate.

### Senescence associated β-galactosidase assay

Cells to be assayed were seeded in 6-well plates for the senescence-associated b-galactosidase assay. After 24 h, cells were fixed and stained with X-gal for detection of b-galactosidase activity using a senescence b-galactosidase staining kit (Cell Signaling, MA). After 24 h incubation, cells exhibiting positive β-galactosidase activity (turned blue) at pH 6.0 were counted under a light microscope. The percentage of cells exhibiting positive staining was determined in 10 individual fields of interest.

### Western Blotting

Western Blotting was performed as described previously [9]. Cells for analysis were harvested by trypsinization, centrifuged at 1000 rpm and washed once with ice-cold PBS buffer. Cells were then lysed using the Total Protein Extraction Kit (EMD Millipore, MA) based on protocol provided by the manufacturer. Cell lysates were then subjected to Western blot analyses using specific antibodies to various cyclins. Cell lysates were prepared from wild type and CDK2AP1 knockdown primary human fibroblasts and analyzed for p53, p21 and β-tubulin expression by Western blot using specific antibodies. p53 and p21 antibodies were obtained from Cell Signaling, MA, p14^ARF^ antibodies were obtained from Santa Cruz Technologies, while β-tubulin antibody was obtained from Developmental Studies Hybridoma Bank, IA. Appropriate infrared emitting-conjugated secondary antibodies were obtained from Invitrogen, CA. Detection was then carried out using the Odyssey Infrared Imaging System (Li-Cor Biosciences, NE).

## Results

### Knockdown of CDK2AP1 in primary human fibroblasts results in decreased cell proliferation and alters cell cycle profile

**Fig 1:**
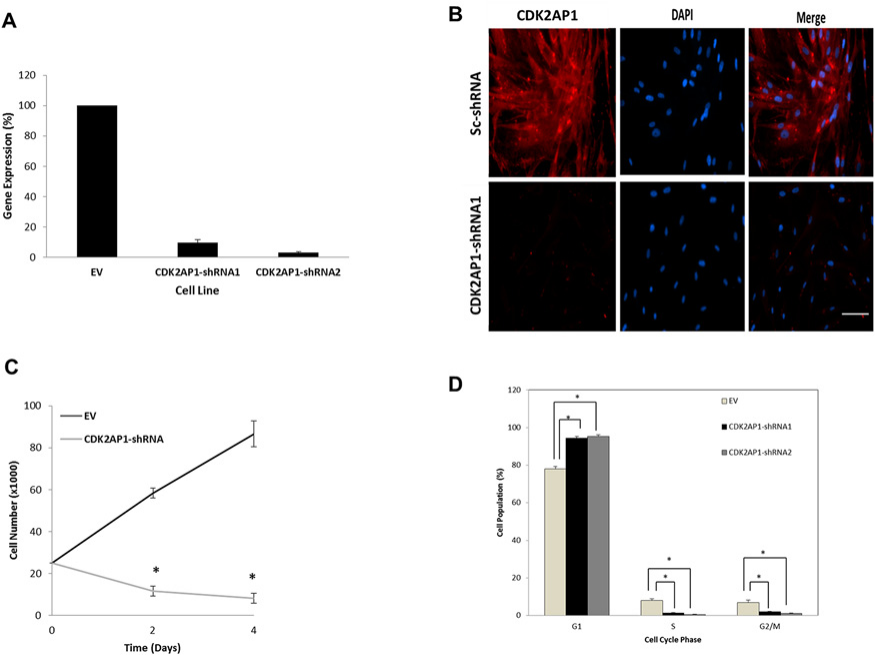
Knockdown of CDK2AP1 in HDFs reduces proliferation and induces G1 phase arrest. Panel A: Primary HDFs were transduced with an empty vector or with CDK2AP1 specific shRNA1 or 2. Following antibiotic selection, cells were examined for *CDK2AP1* expression using qPCR. CDK2AP1 was successfully downregulated using both shRNA’s when compared to EV transduced cells. Panel B: HDFs were transduced with CDK2AP1 shRNA1 or a scrambled-sequence shRNA and stained with a CDK2AP1 specific antibody. Panel C: CDK2AP1 knockdown inhibited proliferation of HDFs. Cells were plated after selection, and counted at different time points by trypan blue exclusion method. Panel D: Asynchronized wild type and CDK2AP1 knockdown HDFs were harvested and stained with propidium iodide. DNA content was analyzed by flow cytometry. Results are presented together with standard deviation from experiments conducted in triplicate.

To investigate the effect of CDK2AP1 knockdown on the growth of primary human fibroblasts, we used lentiviral delivery of shRNA1 to downregulate CDK2AP1 in primary human dermal fibroblasts. CDK2AP1 expression was then examined using qPCR (Fig. 1A) and immunocytochemistry (Fig. 1B). Based on qPCR analysis, we were able to achieve 90% knockdown of CDK2AP1, with statistical analysis indicating significant differences (p <0.05) in the expression patterns between primary human fibroblasts transduced with the shRNA1 and normal fibroblasts. Additional line was created by transducing with a second different CDK2AP1 shRNA (shRNA2) which resulted in a 96% knockdown of CDK2AP1 (Fig. 1A). Following the generation of CDK2AP1 knockdown primary human fibroblasts, we examined the effect of the knockdown on cell proliferation. Initial analyses conducted in triplicate using hemocytometer to count cells, indicated that CDK2AP1 knockdown cells exhibited an extremely slow rate of proliferation, suggesting that depletion of CDK2AP1inhibited cell proliferation (Fig. 1C). Subsequent analysis using the BrdU proliferation assay indicated that the CDK2AP1 knockdown cells had a significantly lower BrdU incorporation than the wild type fibroblasts (p-value = 0.002) (S1 Fig.). Taken together, these results suggest that the knockdown of CDK2AP1 in primary human fibroblasts decreases proliferation.

Cell cycle analysis was then conducted on an equal number of HDFs transduced with an empty vector (EV) or HDFs transduced with CDK2AP1 shRNA1 or HDFs transduced with CDK2AP1 shRNA2. Analysis demonstrated that the knockdown of CDK2AP1 significantly increased cells in the G1 phase of the cell cycle from 78% in the control cells to 94% in CDK2AP1 shRNA1 transduced cells (p-value = 0.00026) to 95% in CDK2AP1 shRNA2 transduced cells (p-value = 0.00022) (Fig. 1D). In addition, knockdown resulted in a significant decrease in the percentage of cells in the S-phase of the cell cycle from 8% in the control cells to 1.3% in the CDK2AP1-shRNA1 transduced cells (p-value = 0.005) and to 0.6% in the cells transduced CDK2AP1-shRNA2 (p-value = 0.004) (Fig. 1D). Under each condition, we also observed few necrotic/apoptotic cells present in the sub G0/G1 phase of the cell cycle with 7% in the control sample, and 3% and 4% in the CDK2AP1-shRNA1 and CDK2AP1-shRNA2 transduced cells, respectively. Taken together, these results indicate that the depletion of CDK2AP1 prevents progression from the G1 to the S phase of the cell cycle.

### Knockdown of CDK2AP1 in primary human fibroblasts induces premature senescence

**Fig 2:**
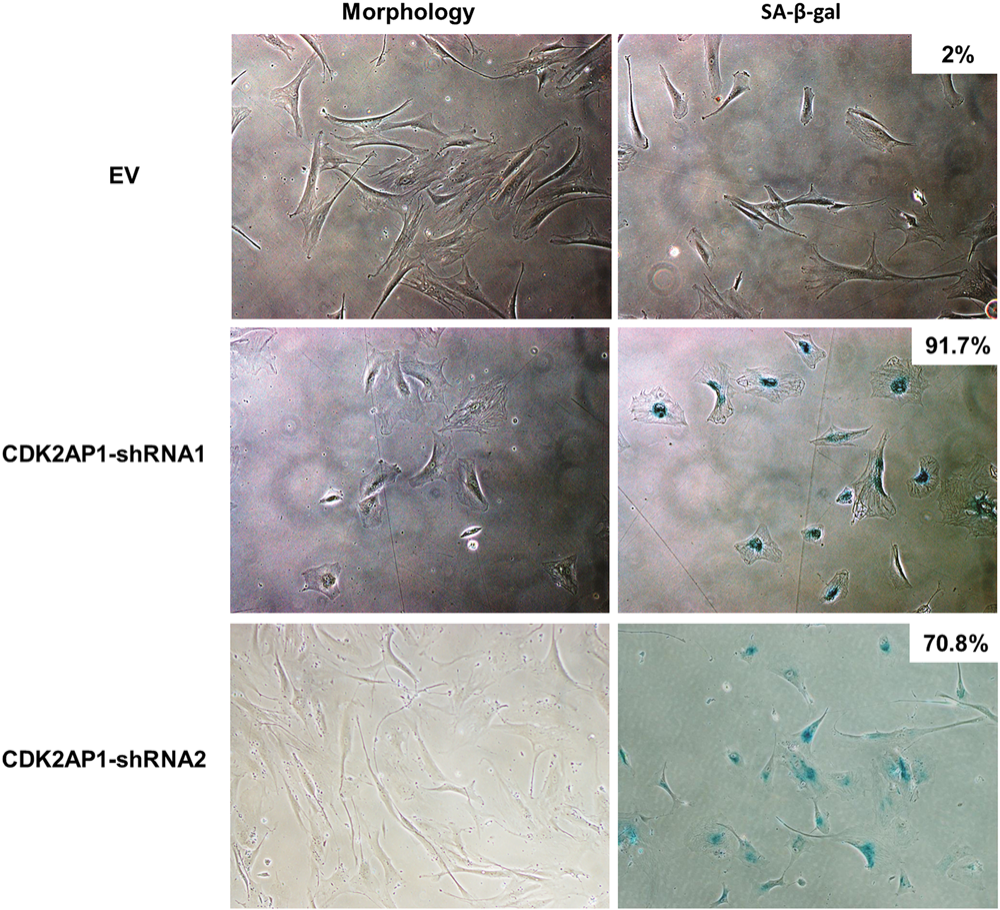
Knockdown of CDK2AP1 altered the morphology of HDFs and increased senescence-associated β-galactosidase activity. HDFs that were transduced with an empty vector (EV) or CDK2AP1-shRNA1 or CDK2AP1-shRNA2, were seeded and grown in a 6-well plate and senescence-associated β-galactosidase assay was performed. CDK2AP1 knockdown fibroblasts displayed a significantly higher senescence associated β-galactosidase activity. The percentage of cells exhibiting β-galactosidase activity is shown at the top of each panel in the right.

During the period of monitoring cell viability, we observed that CDK2AP1 knockdown fibroblasts displayed a flattened cellular morphology with progressive cell passage number. To investigate whether CDK2AP1 knockdown leads to growth arrest, HDFs transduced with an empty vector or HDFs transduced with CDK2AP1-shRNA1 or HDFs transduced with CDK2AP1-shRNA2, were plated for senescence associated β-galactosidase assay (SA-β-gal), typically after 12 days post-transduction. Detection of β-galactosidase activity at pH 6.0 is a known characteristic of senescent cells [11]. We found that 91.7% of HDFs transduced with CDK2AP1-shRNA1 and 70.8% of CDK2AP1-shRNA2 transduced cells displayed appearance of enlarged blue cells in contrast to the control EV transduced cells, which failed to exhibit a detectable blue appearance (Fig. 2). Therefore, these results suggest that knockdown of CDK2AP1 leads primary human fibroblasts to undergo premature senescence.

### Knockdown of CDK2AP1 in primary fibroblasts increases DNA damage

To ascertain the reason behind the unexpected reduced proliferation of the CDK2AP1 knockdown HDFs, we hypothesized that CDK2AP1 knockdown might contribute to a temporary abnormal increase in the progression into S-phase of the cell cycle and hence, an uncontrolled increase in DNA synthesis. This is based on the reported function of CDK2AP1 as a negative regulator of CDK2 and we expected that this could be contributing to errors in DNA synthesis and DNA damage [3].

**Fig 3:**
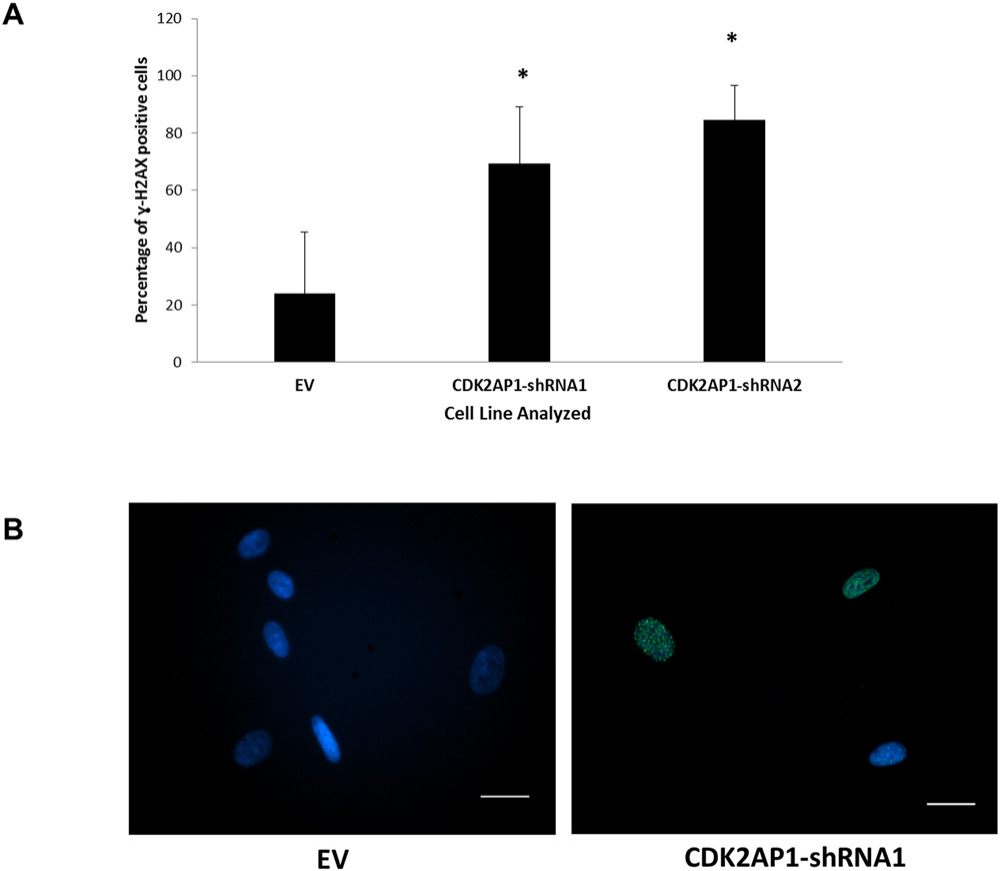
Knockdown of CDK2AP1 in Primary HDFs Increases γ-H2AX Signal. Panel A: Primary HDFs were either transduced with empty vector (EV), CDK2AP1-shRNA1 or CDK2AP1-shRNA2. Following antibiotic selection, immunocytochemistry was carried out using a γ-H2AX specific antibody. Percentage of γ-H2AX was calculated in multiple (n = 28) randomly selected fields by dividing the number of γ-H2AX positive cells by total number of cells in the field. Results are presented together with standard deviation from multiple fields of view (*-p-value < 0.05). Panel B: Example of EV and CDK2AP1-shRNA1 cells stained with γ-H2AX (green) and the nuclear dye, DAPI (blue). Scale bar represents 50 μm.

To examine this possibility, we measured the number of γ-H2AX foci in equal numbers of CDK2AP1-shRNA1, CDK2AP1-shRNA2 or EV transduced cells that were seeded onto 4-chamber slides and stained using γ-H2AX antibody. We found that the knockdown of CDK2AP1 in HDFs resulted in a significant increase in γ-H2AX foci with 70% of the CDK2AP1-shRNA1 HDFs and 84.5% CDK2AP1-shRNA2 cells staining positive, while only 25% of EV cells were positive, indicating a potential increase in DNA damage upon CDK2AP1 knockdown (Fig. 3). Additionally, nuclear staining with DAPI dye indicated that the CDK2AP1-knockdown cells exhibited a high number of micronuclei that were surrounding the nuclei of the cells; a phenomenon not observed in the control fibroblasts (S1 Fig.). This observation might be considered evidence of genomic instability that the knockdown fibroblasts might have experienced before they entered senescence.

### Knockdown of CDK2AP1 increases the levels of p53 and its target genes, *p21*, *BAX* and *PUMA*

**Fig 4:**
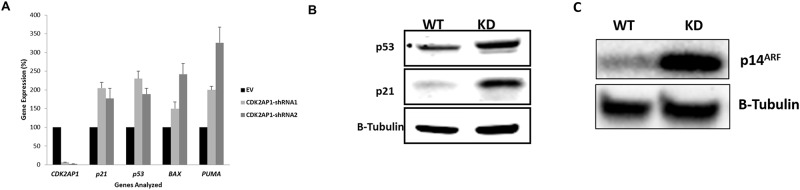
Knockdown of CDK2AP1 increases the expression of p53, p21 and the Apoptotic Genes *BAX* and *PUMA*. Panel A: HDFs transduced with EV, CDK2AP1-shRNA1 or CDK2AP1-shRNA2 were harvested and analyzed for the mRNA expression of *p53*, *p21*, *BAX* and *PUMA*. Knockdown of CDK2AP1 in HDFs increased the expression of these genes. Panel B: Whole cell lysates were extracted from wild and knockdown HDFs and analyzed for p21 and p53 levels by Western Blot. Panel C: Whole cell lysates were extracted from wild and knockdown HDFs and analyzed for p14^ARF^ by Western Blot.

Following CDK2AP1 knockdown in primary HDFs we observed a decrease in cell proliferation, a G1 phase arrest and an increase in cellular senescence. These findings may indicate an increase in the levels of the tumor suppressor p53. Indeed we have found that HDFs in which CDK2AP1 was down regulated had a significant increase in p53 protein levels (Fig. 4). Given that the p21 is a downstream transcriptional target of p53 and plays a role in cell cycle arrest and senescence, we measured its mRNA and protein levels in CDK2AP1 wild type and knockdown HDFs [12]. We found that *p21* mRNA levels increased by 2.1 fold in the CDK2AP1-shRNA1 cells and by 1.8 fold in the CDK2AP1-shRNA2 HDFs when compared with EV transduced cells (Fig. 4A). Western blot analysis further confirmed the increase in p21 protein levels (Fig. 4B).

Due to observed decrease in cell viability upon CDK2AP1 knockdown, we also decided to examine the expression of candidate p53-responsive apoptotic genes, *BAX* and *PUMA*. QPCR analysis indicated that *BAX* mRNA levels were increased by 1.5 fold in the CDK2AP1-shRNA1 transduced HDFs and by 2.4 fold in the CDK2AP1-shRNA2 when compared with EV transduced cells. Similarly, we observed that *PUMA* mRNA levels were increased by 2.0 fold in the CDK2AP1-shRNA1 cells and by 3.6 fold in cells transduced with CDK2AP1-shRNA2 when compared with EV cells.

It is also worth noting that we have observed an increase in *p53* mRNA levels (Fig. 4A) in CDK2AP1 knockdown cells (2.3 fold and 1.89 fold increase in CDK2AP1-shRNA1 HDFs and CDK2AP1-shRNA2 respectively). CDK2AP1 is known to be a member of the Nucleosome Remodeling and Deacetylation (NuRD) Complex [13], which is a repressive complex responsible for epigenetic regulation of numerous genes. Downregulation of members of the NuRD complex has been previously associated with the transcriptional activation of p53 [14]. Therefore, we speculate that the knockdown of CDK2AP1 may have led to aberrant epigenetic regulation of the p53 gene, leading to abnormal increase in its transcription.

### Knockdown of CDK2AP1 Increases p14^ARF^ Protein Levels

P14^ARF^ is an alternative reading frame protein product of the CDKN2A locus which functions as a cell cycle regulator [15], and is activated by oncogenic signals, like excessive inactivation of the retinoblastoma protein (pRB) and increased E2F1 activity [16]. P14^ARF^ is directly activated by the E2F1 transcription factor and directly binds the p53 negative regulator, HDM2 and sequesters it in the nucleolus [15]. By antagonizing HDM2, p14^ARF^ allows p53 transcriptional activity which would lead to decreased cell proliferation and apoptosis.

We thus speculated that knockdown of CDK2AP1 might have caused an oncogenic insult as a result of increased CDK2 activity. This may aberrantly increase E2F1 which would increase the levels of p14^ARF^. Using whole cell lysates from CDK2AP1 wild type and knockdown HDFs, we conducted Western Analysis and observed a significant increase in the protein levels of p14^ARF^ as expected (Fig. 4C). This suggests that the increase in p14^ARF^ maybe the underlying cause of p53 upregulation and the observed phenotype in the CDK2AP1 HDFs.

### Observed Senescence in CDK2AP1 Knockdown Primary HDFs is p53 Dependent

**Fig 5:**
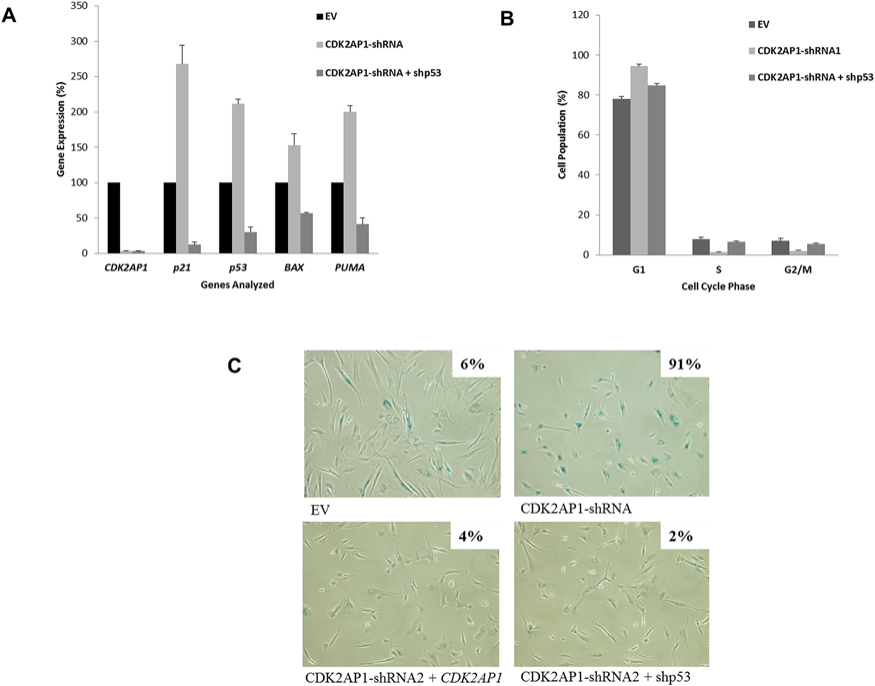
Observed phenotype following CDK2AP1 knockdown is p53 dependent. Panel A: Primary HDFs were transduced with EV or CDK2AP1-shRNA or CDK2AP1-shRNA and a p53 specific shRNA and were analyzed by qPCR for mRNA levels of *p53*, *p21*, *BAX* and *PUMA*. Knockdown of p53 with CDK2AP1 prevented the increase in p21, BAX and PUMA. Panel B: The knockdown of p53 with CDK2AP1 increased the percentage of cells in the S and reduced the cells in G1 when compared to CDK2AP1 only knockdown cells (p-value < 0.05). Results are presented together with standard deviation from experiments conducted in triplicate. Panel C: HDFs that were transduced with an EV, CDK2AP1 shRNA1, CDK2AP1 shRNA2 and exogenous *CDK2AP1* (CDK2AP1-shRNA2+ *CDK2AP1*) or CDK2AP1 shRNA2 and p53 shRNA (CDK2AP1-shRNA2+shp53) were seeded onto a 6-well plate and senescence-associated β-galactosidase assay was performed. Simultaneous knockdown of CDK2AP1 and p53 results in significantly reduced senescence associated β-galactosidase activity. The percentage of cells exhibiting β-galactosidase activity is shown at the top of each panel.

Following CDK2AP1 knockdown, we have observed an increase in p53 levels (Fig. 4). The increase in p53 may have resulted in the observed increase of the transcription of *p21*, *BAX* and *PUMA* (Fig. 4A). To investigate if the observed increase in the levels of transcription of the aforementioned genes in the CDK2AP1 knockdown primary HDFs is p53 dependent, we co-transduced primary HDFs with p53 and CDK2AP1 shRNAs and assayed *CDK2AP1*, *p53*, *p21*, *BAX* and *PUMA* expression by qPCR analysis. The double-knockdown cells exhibited significantly lowered expression of *p53*, *p21*, *BAX* and *PUMA* (p-value <0.05) when compared with CDK2AP1 only knockdown cells (Fig. 5A), thus suggesting that the observed increase in *p21*, *BAX* and *PUMA* transcription levels in the CDK2AP1 only knockdown cells is p53 dependent.

In experiments conducted with primary HDFs, we found that knockdown of CDK2AP1 resulted in increased percentage of cells in the G1 phase of the cell cycle and prevented progression from the G1 to the S phase of the cell cycle (Fig. 1D). To test dependence on p53 expression, we examined the cell cycle profile of primary HDFs that were co-transduced with p53 and CDK2AP1 shRNAs. We found that the knockdown of p53 significantly increased the percentage of cells in the S phase (p-value = 0.0007) and reduced the cells in G1 phase of the cell cycle (p-value = 0.0004) (Fig. 5B) when compared to CDK2AP1 knockdown only primary HDFs (Fig. 1D).

To investigate if the simultaneous knockdown of p53 and CDK2AP1 in primary HDFs would prevent premature senescence, HDFs transduced with an empty vector or HDFs transduced with CDK2AP1 shRNA1 or HDFs transduced with CDK2AP1 shRNA2 and exogenous *CDK2AP1* (CDK2AP1-shRNA2 + *CDK2AP1*) or HDFs transduced with CDK2AP1 shRNA2 and p53 shRNA (CDK2AP1-shRNA2 + shp53) were plated for senescence associated β-galactosidase assay (SA-β-gal). Cells were fixed and stained with X-gal for the detection of β-galactosidase activity (Fig. 5C). Our results indicate that the fibroblasts that were co-transduced with the CDK2AP1 and p53 shRNAs did not enter premature senescence and had extremely low β-galactosidase activity. Taken together, these results indicate that downregulation of CDK2AP1 increases p53 expression which pushes primary human fibroblasts to enter a p53 dependent premature senescence.

## Discussion

The function of CDK2AP1 as initially assessed in multiple cancer cell lines indicates its role in regulation of apoptosis, proliferation and invasion [2]. Although the primary function of CDK2AP1 as a cell cycle regulator that controls the G1-S phase transition by negatively regulating CDK2 has been ascertained [1], its role in primary human fibroblasts has not been studied. To investigate the function of CDK2AP1 in primary human fibroblasts, we knocked down CDK2AP1 expression in primary human dermal fibroblasts (HDFs) using short hairpin RNA. Using a lentiviral approach, we were able to downregulate CDK2AP1 by ~90%. Following knockdown of CDK2AP1 in primary HDFs, we observed a distinct reduction in the proliferation potential of these cells, with the cells exhibiting morphological changes as well. The CDK2AP1 knockdown HDFs appeared enlarged, flat and more spread out. Given that reduced proliferation and flattened morphologies is a sign of premature senescence in primary somatic cells, we decided to examine the CDK2AP1 knockdown HDFs for senescence associated β-galactosidase activity. Results indicated that CDK2AP1 knockdown HDFs displayed a significantly higher senescence associated β-galactosidase activity.

Given that CDK2AP1 is a known inhibitor of CDK2 and has also been reported to associate with the DNA polymerase/alpha primase complex [1,3], we expected that following its knockdown, the inhibition might have been alleviated allowing abnormal increase in DNA synthesis, which could lead to DNA damage. Using immunocytochemical analysis, we examined the CDK2AP1 knockdown cells for levels of γ-H2AX foci, a known indicator of DNA damage. Our results indicated that the CDK2AP1 knockdown HDFs had significantly higher number of cells that were γ-H2AX positive (having more than three γ-H2AX foci in each cell). Additionally, we observed that the CDK2AP1 knockdown HDFs had a high number of micronuclei that were surrounding the nuclei when examined by DAPI staining (S2 Fig.). These findings might be considered evidence of genomic instability that the CDK2AP1 knockdown HDFs might have experienced before they entered senescence.

Also, following CDK2AP1 knockdown, an increase in CDK2 activity may have occurred, causing an abnormal increase in the phosphorylation of the pRB protein and a deregulation in the G1-S phase transition. A previous study demonstrated that *Cdk2ap1* deletion indeed resulted in hyperphosphorylation of pRB [18]. Hyperphosphrylation of the pRB protein would increase the activity of the transcription factor E2F1 which in turn, would bind the promoter of p14^ARF^ and induce an increase in its levels. We have found that CDK2AP1 knockdown in primary HDFs resulted in a prominent increase in the levels of p14^ARF^ protein. As mentioned earlier, p14^ARF^ antagonizes HDM2 and promotes an increase in p53 levels [16]. Therefore, we expect that the observed phenotype is a result of p14^ARF^-mediated increase in p53 activity. The role of p14^ARF^ in response to DNA damage has also been demonstrated in a recent study, with results indicating that that persistent DNA damage increases the levels of p14^ARF^ and that the increase is ATM dependent [19]. P14^ARF^ then allows p53 transcription of the phosphatases DUSP4 and DUSP7, which in turn dephosphorylates ERK1 and inhibit cell proliferation. Therefore, in future experiments, it would be useful to attempt to reverse the senescence observed in CDK2AP1 knockdown primary human fibroblasts by downregulating p14^ARF^ or ATM, given that the DNA damage response (DDR) pathway via ATM activation and ARF are stress responders and activate p53, while ATM also keeps in check ARF activation [20,21].

Although the majority of existing reports support the role of CDK2AP1 to be a tumor suppressor, recent studies have shown that knockdown of CDK2AP1 in human glioma cells reduced proliferation, caused a G0/G1 cell cycle arrest and increased apoptosis [7]. Following CDK2AP1 knockdown in primary human fibroblasts, we have also observed a reduction in proliferation, a G0/G1 arrest and an increase in p21 and the candidate p53-responsive apoptotic genes *BAX* and *PUMA* when examined by qPCR (Fig. 4).

Given that CDK2AP1 acts as a regulator of G1 to S phase progression by inhibiting CDK2, knockdown of CDK2AP1could aberrantly increase the activity of E2F1 through pRB inactivation. By being a downstream transcriptional target of E2F, p14^ARF^ is activated by oncogenic signals, and through modulation of MDM2, promotes the increase in p53 levels [16,17]. To our knowledge there are no studies that prove that the deletion of CDK2AP1 alone can transform cells. Therefore we speculate that the ability of a deleterious mutation in CDK2AP1 to transform cells is highly dependent on the presence of an additional mutation in other tumor suppressor like p21, p27, p14^ARF^ or p53. In the study conducted with human gastric cancer tissues (n = 180), it was determined that p53 expression was negative in 95 (54%), while CDK2AP1 expression was negative in 140 (77%), with no apparent correlation found between CDK2AP1 and p53 expressions. However, it is important to note that the study only examined the levels of CDK2AP1 and p53. Detection and analysis of other mutations in the context of negative expression of CDK2AP1 is worthy of further study.

Downregulation of CDK2AP1 in cancer cells could thus result in beneficial growth inhibitory effects. This would only be possible if the tumor suppressors or the genes involved in the pathway activated by CDK2AP1 knockdown which causes growth inhibition were normal and not mutated in the cancer cell line of interest. Therefore, further studies are needed to identify the pathway responsible for the observed anti-tumorigenic effects of CDK2AP1 knockdown, so its potential can be examined in the appropriate cancer cell lines.

CDK2AP1 has also been reported to be a member of the epigenetically repressive complex, NuRD [13]. A previous study demonstrated that disruption of a member of the NuRD complex leads to an increase in p53 levels [14]. In our studies, we have observed an increase in *p53* transcription following CDK2AP1 knockdown, which could be a result of the disruption of the NuRD complex. Deletion of CDK2AP1 has been previously reported to alter epigenetic marks on genes that are regulated by the NuRD complex, and these genes could not be silenced in the absence of CDK2AP1 due to disrupted localization of NuRD [22]. Therefore, it is possible that following CDK2AP1 knockdown, the NuRD complex could not localize properly to regulate *p53* transcription, which could thus have caused the observed increased levels of *p53* in the CDK2AP1 knockdown HDFs.

Given the observed increase in p53 levels upon CDK2AP1 knockdown, we decided to extensively examine the dependence on p53 in the CDK2AP1 knockdown HDFs. We observed that when p53 and CDK2AP1 were simultaneously downregulated in primary HDFs, the cells did not enter senescence based on reduced senescence associated β-galactosidase activity. The simultaneous knockdown of p53 and CDK2AP1 also led to reduction of accumulation of cells in the G1 phase and increased cells in the S phase of the cell cycle. qPCR analysis further demonstrated that knockdown of p53 in HDFs prevents the CDK2AP1 knockdown induced increase in *p21*, *BAX* or *PUMA* gene expression. Together, these results lead us to conclude that the CDK2AP1knockdown induced senescence in primary HDFs is p53 dependent.

## Supporting Information

S1 Fig Knockdown of CDK2AP1 in primary HDFs reduces BrdU incorporation. A. Cells transduced with an empty vector (EV) or CDK2AP1-shRNA1 were labeled with 20 μM BrdU for 2 h, fixed and stained with a BrdU specific antibody and the percentage of BrdU positive cells was calculated. Results are presented together with standard deviation from experiments conducted in triplicate. (*-p-value < 0.05). B. Showing representative pictures of the quantified immunocytochemistry. BrdU positive cells are shown in red, nuclei were stained with DAPI (blue). Scale bar represent 50 μm.
(TIF)

S2 Fig Knockdown of CDK2AP1 in primary HDFs leads to increased micronuclei formation. Red arrows point to micronuclei, which may be a sign of genetic instability in these cells. Scale bar represents 100 μm.
(TIF)

S1 Table Sequences of primers used in qPCR analysis. (DOC)
