# Supplementary figures and images for: Knockdown of CDK2AP1 in Primary Human Fibroblasts Induces p53 Dependent Senescence
Source: PLoS One. 2015 Mar 18;10(3):e0120782. doi: 10.1371/journal.pone.0120782 (PMC4365013; doi:10.1371/journal.pone.0120782)

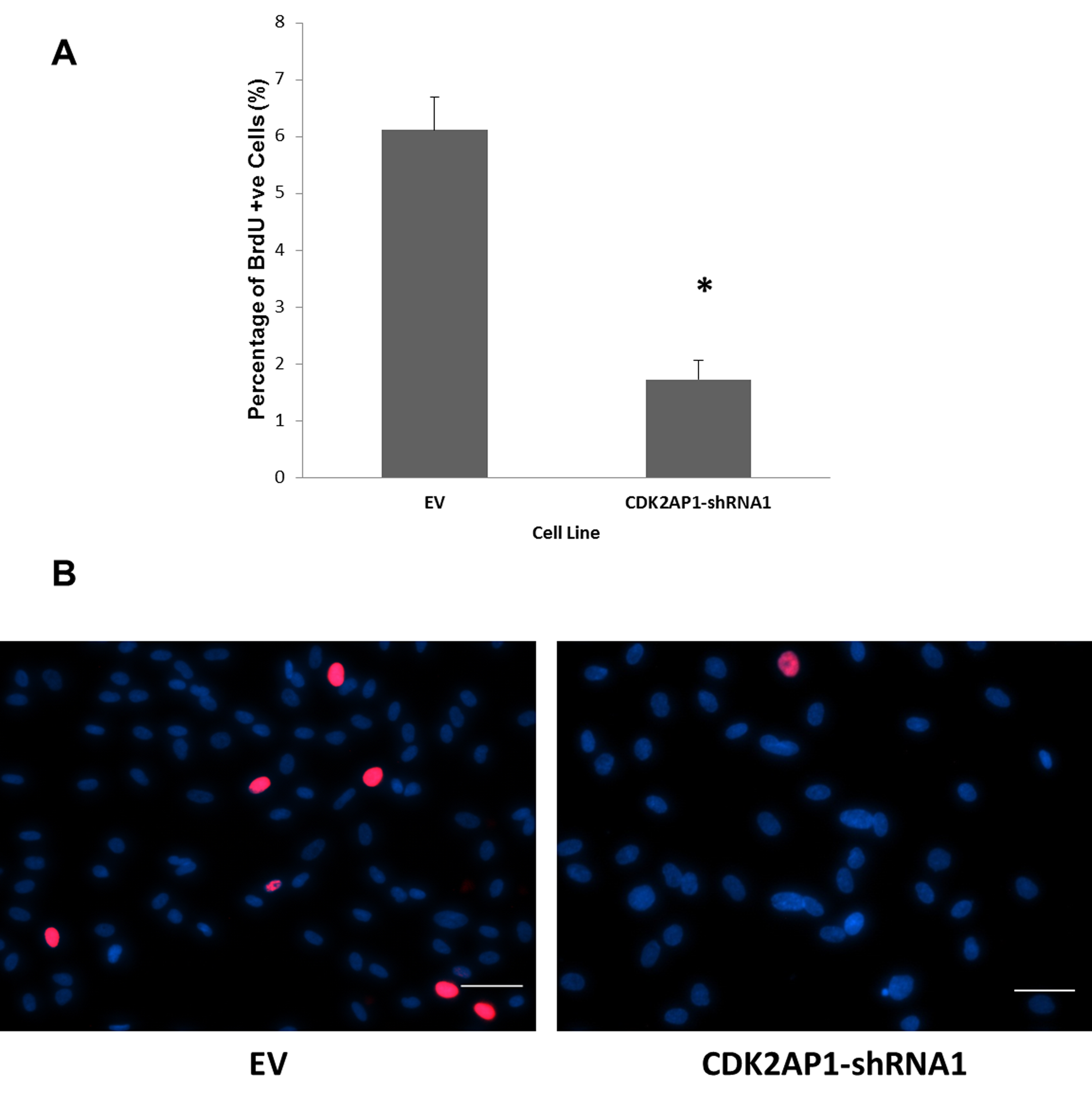

Supplement: S1 Fig — A. Cells transduced with an empty vector (EV) or CDK2AP1-shRNA1 were labeled with 20 μM BrdU for 2 h, fixed and stained with a BrdU specific antibody and the percentage of BrdU positive cells was calculated. Results are presented together with standard deviation from experiments conducted in triplicate. (*-p-value < 0.05). B. Showing representative pictures of the quantified immunocytochemistry. BrdU positive cells are shown in red, nuclei were stained with DAPI (blue). Scale bar represent 50 μm. (TIF) [file pone.0120782.s001.tif]

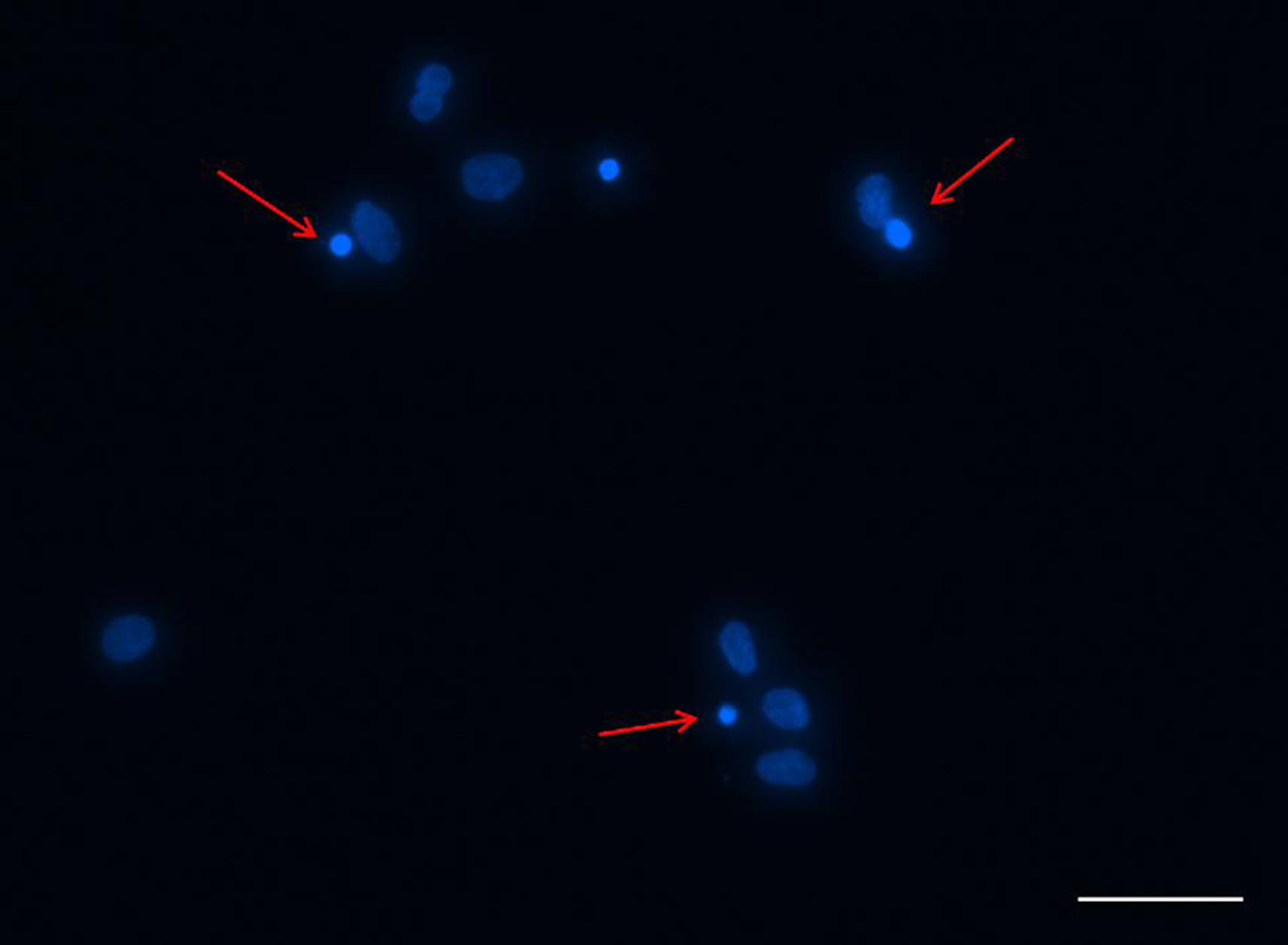

Supplement: S2 Fig — Red arrows point to micronuclei, which may be a sign of genetic instability in these cells. Scale bar represents 100 μm. (TIF) [file pone.0120782.s002.tif]
