# Supplementary material for: Knockdown of CDK2AP1 in Primary Human Fibroblasts Induces p53 Dependent Senescence
Source: PLoS One. 2015 Mar 18;10(3):e0120782. doi: 10.1371/journal.pone.0120782 (PMC4365013; doi:10.1371/journal.pone.0120782)
Supplement: S1 Table — (DOC) [file pone.0120782.s003.doc]

**Tables**

**Supplementary Table 1:** Sequences of primers used in qPCR analysis**.**

| **Primer** | **Sequence** |
| --- | --- |
| GAPDH Forward | TTGCCATCAATGACCCCTTCA |
| GAPDH Reverse | CGCCCCACTTGATTTTGGA |
| CDK2AP1 Forward | ATGTCTTACAAACCGAACTTGGC |
| CDK2AP1 Reverse | GCCCGTAGTCACTGAGCAG |
| CDKN1A (p21) Forward | TGTCCGTCAGAACCCATGC |
| CDKN1A (p21) Reverse | AAAGTCGAAGTTCCATCGCTC |
| Tp53 Forward | CAGCACATGACGGAGGTTGT |
| Tp53 Reverse | TCATCCAAATACTCCACACGC |
| PUMA Forward | GACCTCAACGCACAGTACGAG |
| PUMA Reverse | AGGAGTCCCATGATGAGATTGT |
| BAX Forward | CCCGAGAGGTCTTTTTCCGAG |
| BAX Reverse | CCAGCCCATGATGGTTCTGAT |
